# Role of postoperative chemoradiotherapy in head and neck cancer without positive margins or extracapsular extension: a propensity score-matching analysis

**DOI:** 10.1186/s13014-022-02152-w

**Published:** 2022-11-13

**Authors:** Zhi-Qiao Liu, Pu-Yun OuYang, Bao-Yu Zhang, En-Ni Chen, Su-Ming Xiao, Shan-Shan Yang, Zhong-Yuan Yang, Fang-Yun Xie

**Affiliations:** 1 Department of Radiation Oncology, Sun Yat-Sen University Cancer Center, State Key Laboratory of Oncology in South China, Collaborative Innovation Center for Cancer Medicine, 651 Dongfeng Road East, Guangzhou, Guangdong People’s Republic of China; 2 Department of Head and Neck Cancer, Sun Yat-Sen University Cancer Center, State Key Laboratory of Oncology in South China, Collaborative Innovation Center for Cancer Medicine, Guangzhou, People’s Republic of China

**Keywords:** Head and neck cancer, Intermediate-risk features, Postoperative chemoradiotherapy, Postoperative radiotherapy, Survival

## Abstract

**Background:**

The aim of this work was to determine whether patients with intermediate-risk head and neck squamous cell carcinoma (HNSCC) can benefit from postoperative chemoradiotherapy (POCRT).

**Methods:**

Patients without extracapsular extension (ECE) or positive margins (PMs) who received POCRT or postoperative radiotherapy (PORT) at our center were retrospectively (December 2009 to October 2018) included for analysis, in particular, using a propensity score-matching method.

**Results:**

After matching, 264 patients were enrolled, including 142 (41.2%) patients with pT3-4, 136 (38.3%) patients with pN2-3, 68 (21.1%) patients with perineural invasion, and 45 (12.8%) patients with lymphatic/vascular space invasion. With a median follow-up of 52 months, 3-year overall survival (OS), locoregional relapse-free survival (LRFS), distant metastasis-free survival (DMFS) and disease-free survival (DFS) rates were 72.4%, 79.3%, 83.5% and 62.5%, respectively. pN2-3 was an independent risk factor for OS (*p* < 0.001), DFS (*p* < 0.001), LRFS (*p* < 0.001) and DMFS (*p* = 0.002), while pT3-4 was a poor prognostic factor for DMFS (*p* = 0.005). Overall, patients receiving POCRT had no significant differences from those receiving PORT in OS (*p* = 0.062), DFS (*p* = 0.288), LRFS (*p* = 0.076) or DMFS (*p* = 0.692). But notably, patients with pN2-3 achieved better outcomes from POCRT than PORT in 3-year OS (*p* = 0.050, 63.9% vs. 47.9%) and LRFS (*p* = 0.019, 74.6% vs. 54.9%). And patients with pT3-4 also had higher 3-year LRFS (*p* = 0.014, 88.5% vs. 69.1%) if receiving POCRT.

**Conclusions:**

Among all intermediate-risk pathological features, pN2-3 and pT3-4 were independent unfavorable prognostic factors for patients with HNSCC without PMs or ECE. POCRT can improve the survival outcomes of patients with pN2-3 or pT3-4.

## Background

According to the latest cancer statistics, head and neck cancer is the eighth most prevalent cancer worldwide with approximately 54,000 new cases and 11,230 deaths in the United States [1]. In China, the situation is worse. There were 77,900 new cases and 40,100 death cases according to the latest cancer statistics [2]. Squamous cell carcinoma is the major pathological type [3]. Surgery-based comprehensive treatment is the standard for resectable locally advanced mucosal head and neck squamous cell carcinoma (HNSCC) [4]. Postoperative chemoradiotherapy (POCRT) or postoperative radiotherapy (PORT) is the typical adjuvant strategy for patients with adverse pathological features. The European Organization for Research and Treatment of Cancer (EORTC) trial no. 22931 [5] enrolled patients arising from oral cavity, oropharynx, larynx or hypopharynx with a wide range of risk factors, including pT3-4, pN2-3, extracapsular extension (ECE), positive margins (PMs), perineural invasion (PNI) and lymphatic/vascular space invasion (LVI), and reported that POCRT achieved higher 5-year progression-free survival and overall survival (OS) than PORT. Meanwhile, the Radiation Therapy Oncology Group (RTOG) trial no. 95-01 [6] was conducted in patients with oral cavity, oropharynx, larynx or hypopharynx squamous cell carcinoma with characteristics of ECE, PMs or two or more regional lymph nodes, and indicated that patients with these factors could achieve better 2-year disease-free survival (DFS) but not OS from POCRT than PORT. Subsequent pooled analysis of these two clinical trials [7] suggested that ECE and PMs were the only two risk factors from which patients benefited significantly from POCRT. Interestingly, Trifiletti et al. [8] found that POCRT could also improve the OS of patients with multiple positive lymph nodes, despite the absence of PMs and ECE. Obviously, except for pT3-4 and pN2-3, other intermediate-risk features, such as LVI and PNI were not taken into consideration due to the lack of data, and it was unknown whether the OS benefit derived from better local or distant control.

Hence, our study aimed to validate the survival benefit of POCRT in patients with multiple lymph nodes when margin was negative and ECE was absent, and explore the effect of POCRT on survival in patients with intermediate-risk features.

## Methods

This retrospective study was approved by the Sun Yat-sen University Cancer Center Institutional Review Board (no. B2019-053) and individual informed consent was waived as the study only required anonymous analysis of routine data. All clinical investigations were conducted according to the Declaration of Helsinki. A total of 337 patients were finally enrolled according to the following inclusion criteria: (i) previously untreated, histologically proven squamous cell carcinoma arising from the oral cavity, oropharynx, larynx or hypopharynx between 30 December 2009 and 31 October 2018; (ii) confirmed no distant metastasis before treatment; (iii) underwent primary surgery performed with curative intent at our cancer center; (iv) had no pathologically positive margin or extracapsular extension; (v) had pathologically proven adverse features, such as LVI, PNI, pT3-4 or pN2-3; and (vi) received POCRT or PORT at our cancer center. The exclusion criteria were as follows: (i) received anticancer therapy elsewhere before the diagnosis; (ii) pregnancy or lactation; (iii) diagnosed with another sort of cancer before or during the treatment, or during the follow-up; and (iv) loss of up within 6 months from treatment. The routine pretreatment workup consisted of complete history taking, physical examination, hematology and biochemistry profiles, computed tomography (CT) or magnetic resonance imaging (MRI) of the head and neck, chest radiography, abdominal sonography, bone scanning and/or positron emission tomography (PET)-CT. In this study, all patients were restaged based on the American Joint Committee on Cancer Staging Manual of Head and Neck Cancer (8th edition) by two doctors to minimize heterogeneity.

Patients were treated depending on their diagnosis, general health condition and the clinicians’ discretion. All patients received intensity-modulated radiotherapy. After image registration, we used postoperative CT and MR images for delineation while pretreatment CT or MRI scans served as reference images of the primary tumor. Delineation of target volume was in accordance with the International Commission on Radiation Units and Measurements reports 50, 62 and 83. The tumor bed of the radiotherapy target was delineated basing on the description of surgery process and radiological images before and after surgery. The clinical target volume (CTV) extended 5–10 mm beyond the tumor bed and the potential path of invasion. As for pathologically positive cervical lymph nodes, a certain cervical region was included in the CTV. The planning target volume (PTV) was a 3 mm extension of the CTV. The PTV of the tumor bed and/or the involved lymph nodes was generally given as a total dose of 60–66 Gy or higher in 30–33 fractions. Platinum-based concurrent chemotherapy was administrated to some patients, including cisplatin (80–100 mg/m^2^), carboplatin (area under the curve of 5), nedaplatin (80–100 mg/m^2^) or oxaliplatin (130 mg/m^2^) every 3 weeks for two to three cycles. In the end, all patients completed the treatment course in both the PORT and POCRT groups.

Patients were followed up with a workup consisting of head and neck CT or MRI, chest radiography, abdominal sonography every 3–6 months for the first 3 years and subsequently every 6–12 months. Bone scan and PET/CT were not routinely conducted at the follow-up examination. Treatment failures were proven by pathological report. If pathological biopsy was not applicable, radiology examination served as an alternative. Salvage treatment, including chemotherapy, surgery, radiotherapy and/or immunotherapy were delivered to patients with local and/or distant treatment failures.

The characteristics of patients were compared using a Student’s *t*-test (continuous variables) or chi-squared test (categorical variables). Survival endpoints included OS (time from treatment to death from any cause), DFS (time from treatment to evidence of disease progression or death from any cause), LRFS (time from treatment to the first locoregional relapse) and DMFS (time from treatment to the first distant metastasis). Survival rates were estimated with Kaplan–Meier method [9] and compared to a log-rank test. Independent effects of variables were evaluated using a Cox proportional hazards model [10]. A propensity score-matching (PSM) method was used to minimize the differences of patients between POCRT and PORT in sex, age, differentiated histopathological type, PNI, LVI, pT3-4, pN2-3 and overall stage at the ratio of 1:1 with the nearest neighbor method.

Statistical analysis was performed using SPSS, version 22.0 (SPSS Inc., Chicago, IL, USA) and R software (version 4.1.3, http://www.rproject.org/). A two-sided *p*-value < 0.05 was considered as significant.

## Results

### Patients’ characteristics

A total of 337 patients with resected mucosal HNSCC were enrolled, of which 88.7% (299/338) were men, 59.9% (202/338) were under 60 years old and 80.4% (271/388) were locally advanced cases. According to the postoperative pathological reports, 142 patients (41.2%) were staged as pT3-4, 136 patients (38.3%) were staged as pN2-3, 68 patients (21.1%) had PNI and 45 patients (12.8%) had LVI. The distribution of primary sites was as follows: oral cavity (42.4%, 143/337), larynx (38.6%, 130/337), hypopharynx (11.3%, 38/337) and oropharynx (8.0%, 27/337).

Initially, significant differences were observed in the N classification (*p* = 0.007) and overall stage (*p* = 0.002) between the two groups. After matching, 132 patients in the POCRT group were matched with 132 patients in the PORT group with balanced baseline characteristics (all *p* ≥ 0.05) (Table 1). In total, there were 93 patients suffering from local, regional and/or distant treatment failure, including 39 local recurrent cases, 38 regional recurrent cases and 47 metastatic cases. A total of 39.7% of the patients with failure received salvage treatments: 20 patients with surgery, 12 with chemotherapy, 4 with radiation and 1 with immunotherapy.

**Table 1:** Clinical characteristics of POCRT and PORT groups in the entire cohort (n = 337) and Matched cohort (n = 264)

Parameters	Entire cohort			Matched cohort		
	PORT (%)	POCRT (%)	*p* value	PORT (%)	POCRT (%)	*p* value
Sex			0.231			1.000
Male	157(86.7)	142(91.0)		117(88.6)	118(89.4)	
Female	24(13.3)	14(9.0)		15(11.4)	14(10.6)	
Age			0.265			0.796
> 60	78(43.1)	57(36.5)		47(35.6)	44(33.3)	
≤ 60	103(56.9)	99(63.5)		85(64.4)	88(66.7)	
Differentiated type			0.588			0.336
Well	48(26.5)	38(24.4)		39(29.5)	34(25.8)	
Moderately	85(47.0)	82(52.6)		60(45.5)	72(54.5)	
Poorly	48(26.5)	36(23.1)		133(25.0)	26(19.7)	
pT3-4			0.580			0.530
Yes	72(39.8)	67(42.9)		50(37.9)	56(42.4)	
No	109(60.2)	89(57.1)		82(62.1)	76(57.6)	
pN2-3			0.007			0.706
Yes	57(31.5)	72(46.2)		54(40.9)	50(37.9)	
No	124(68.5)	84(53.8)		78(59.1)	82(62.1)	
PNI			0.424			0.271
Yes	35(19.3)	36(23.1)		21(15.9)	29(22.0)	
No	146(80.7)	120(76.9)		111(84.1)	103(78.0)	
LVI			0.330			1.000
Yes	20(11.0)	23(14.7)		14(10.6)	13(9.8)	
No	161(89.0)	133(85.3)		118(89.4)	119(90.2)	
Stage			0.002			0.137
I–II	47(26.0)	19(12.2)		27(20.5)	17(12.9)	
III–IV	134(74.0)	137(87.8)		105(79.5)	115(87.1)	

*POCRT* postoperative chemoradiotherapy; *PORT* postoperative radiotherapy; *PNI* perineural invasion; *LVI* lymphvascular invasion

### Univariate and multivariate survival analysis

With a median follow-up duration of 52 months (range, 0–137 months), the 3- year and 5-year survival rates for the matched cohort were as follows: OS, 72.4% and 64.7%; LRFS, 79.3% and 75.1%; DMFS, 83.5% and 81.7%; and DFS, 62.5% and 56.8%, respectively.

**Fig. 1:**
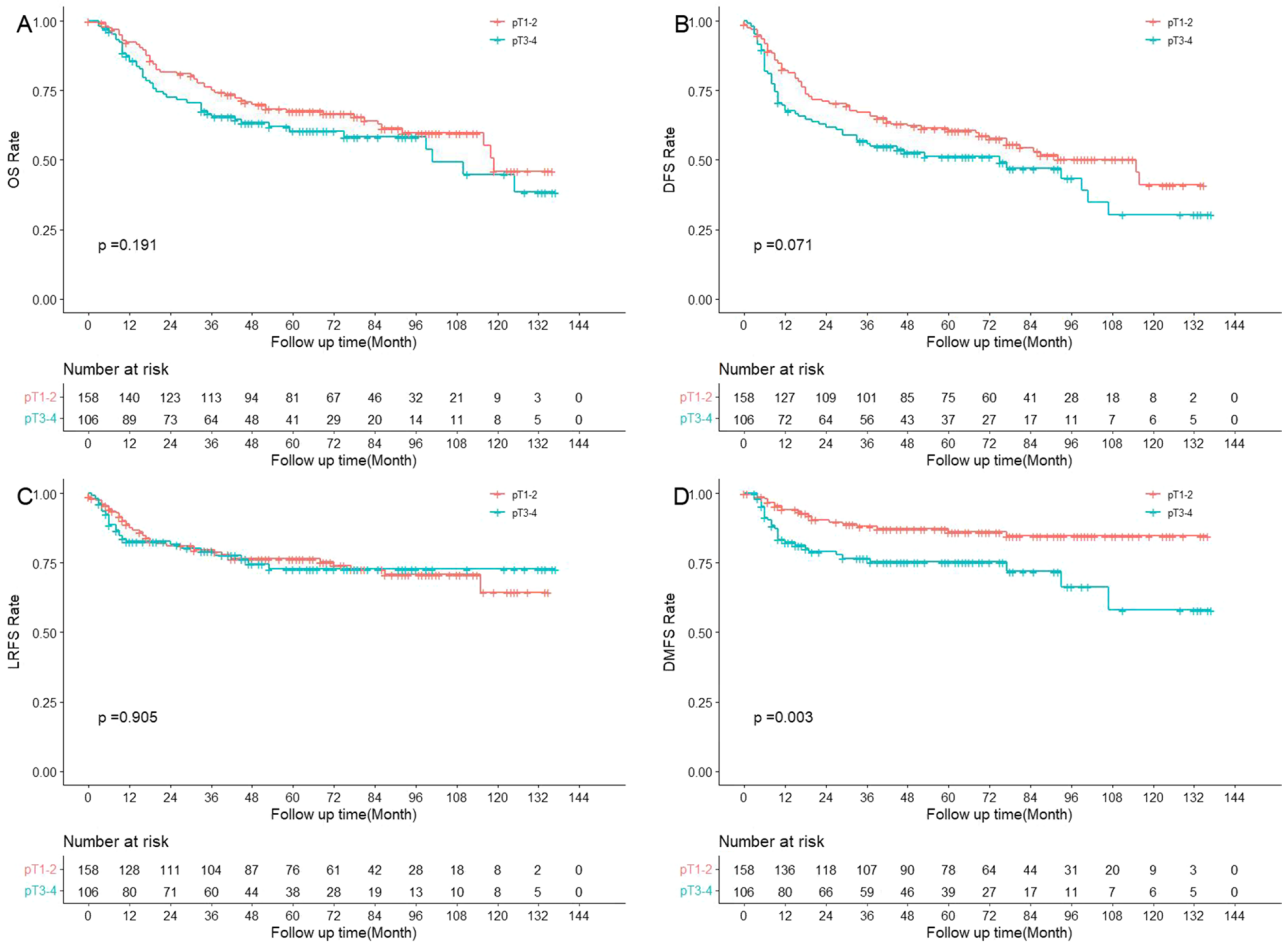
The survival curves of pT1-2 versus pT3-4 in the matched cohort (N = 264)

**Fig. 2:**
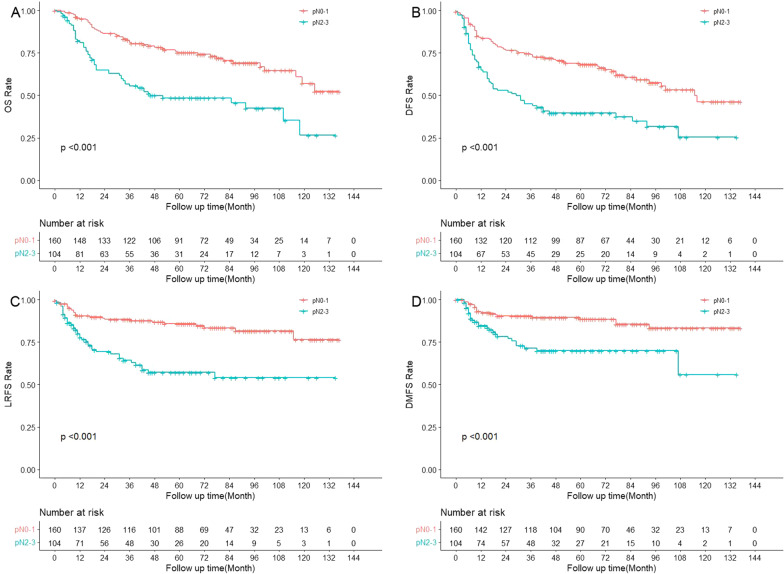
The survival curves of pN0-1 versus pN2-3 in the matched cohort (N = 264)

Univariate survival analysis (Table 2) showed that patients with pT3-4 had worse DMFS than those with pT1-2 (*p* = 0.003, 3-year rate 76.6% vs. 88.1%) (Fig. 1). Patients with pN2-3 had worse survival outcomes than those with pN0-1 in OS (*p* < 0.001, 3-year rate 55.6% vs. 82.5%), DFS (*p* < 0.001, 3-year rate 44.9% vs. 73.9%), LRFS (*p* < 0.001, 3-year rate 64.2% vs. 88.3%) and DMFS (*p* < 0.001, 3-year rate 71.4% vs. 90.3%) (Fig. 2). LVI and PNI were not significantly associated with survival outcomes, including OS, DFS, LRFS or DMFS (all *p* > 0.05). Patients with stage III/IV disease had worse survival outcomes in OS (*p* = 0.001, 3-year rate 67.4% vs. 97.7%), DFS (*p* = 0.002, 3-year rate 57.8% vs. 86.2%), LRFS (*p* = 0.042, 3-year rate 77.4% vs. 88.2%) and DMFS (*p* = 0.002, 3-year rate 80.3% vs. 97.7%) when compared to patients with stage I/II disease.

**Table 2:** The multivariate survival analysis of prognostic factors in the matched cohort

Parameters	HR	95%CI	*p* value
OS			
pN2-3	2.310	1.469–3.632	< 0.001
DFS			
pN2-3	2.346	1.556–3.536	< 0.001
pT3-4	1.527	1.026–2.273	0.037
LRFS			
pN2-3	3.527	1.925–6.461	< 0.001
DMFS			
pN2-3	2.820	1.484–5.357	0.002
pT3-4	2.460	1.306–4.632	0.005

*HR* hazard ratio; *CI* confidence interval; *OS* overall survival; *DFS* disease-free survival; *LRFS* locoregional relapse free survival; *DMFS* distant metastasis free survival

Four adverse pathological features, including T classification, N classification, PNI and LVI were then included in the multivariate analysis. As a result, pN2-3 was still an independent risk factor for OS (*p* < 0.001, hazard ratio (HR) 2.310, 95% confidence interval (CI) 1.469–3.632), DFS (*p* < 0.001, HR 2.346, 95% CI 1.556–3.536), LRFS (*p* < 0.001, HR 3.527, 95% CI 1.925–6.461) and DMFS (*p* = 0.002, HR 2.820, 95% CI 1.484–5.357), whereas pT3-4 was an independent risk factor for DMFS (*p* = 0.005, HR 2.460, 95% CI 1.306–4.632).

### Survival impact of POCRT

**Fig. 3:**
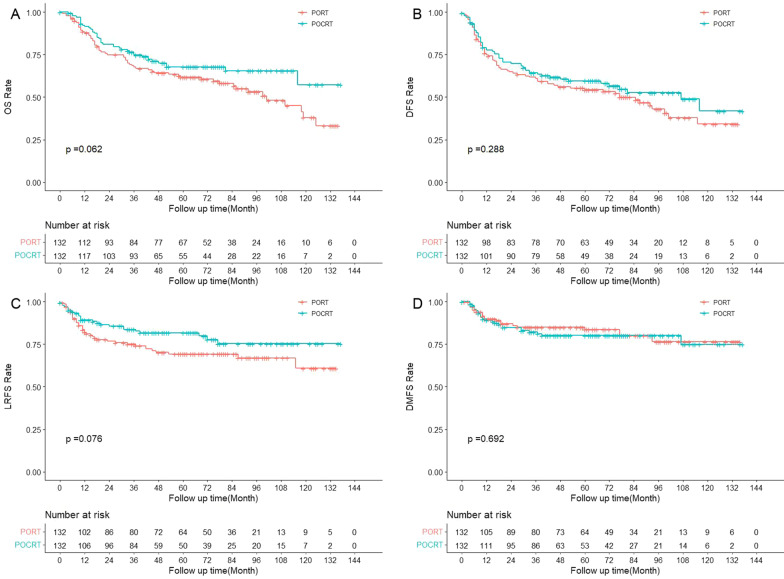
The survival curves of PORT versus POCRT in the matched cohort (N = 264)

In the matched cohort, patients receiving POCRT had no significant survival difference from those receiving PORT, with a 3-year OS rate of 76.4% vs. 67.6% (*p* = 0.062), 3-year DFS rate of 64.2% vs. 60.8% (*p* = 0.288), 3-year LRFS rate of 83.7% vs. 74.0% (*p* = 0.076) and 3-year DMFS rate of 82.1% vs. 85.0% (*p* = 0.692) in the univariate survival analysis (Fig. 3).

**Fig. 4:**
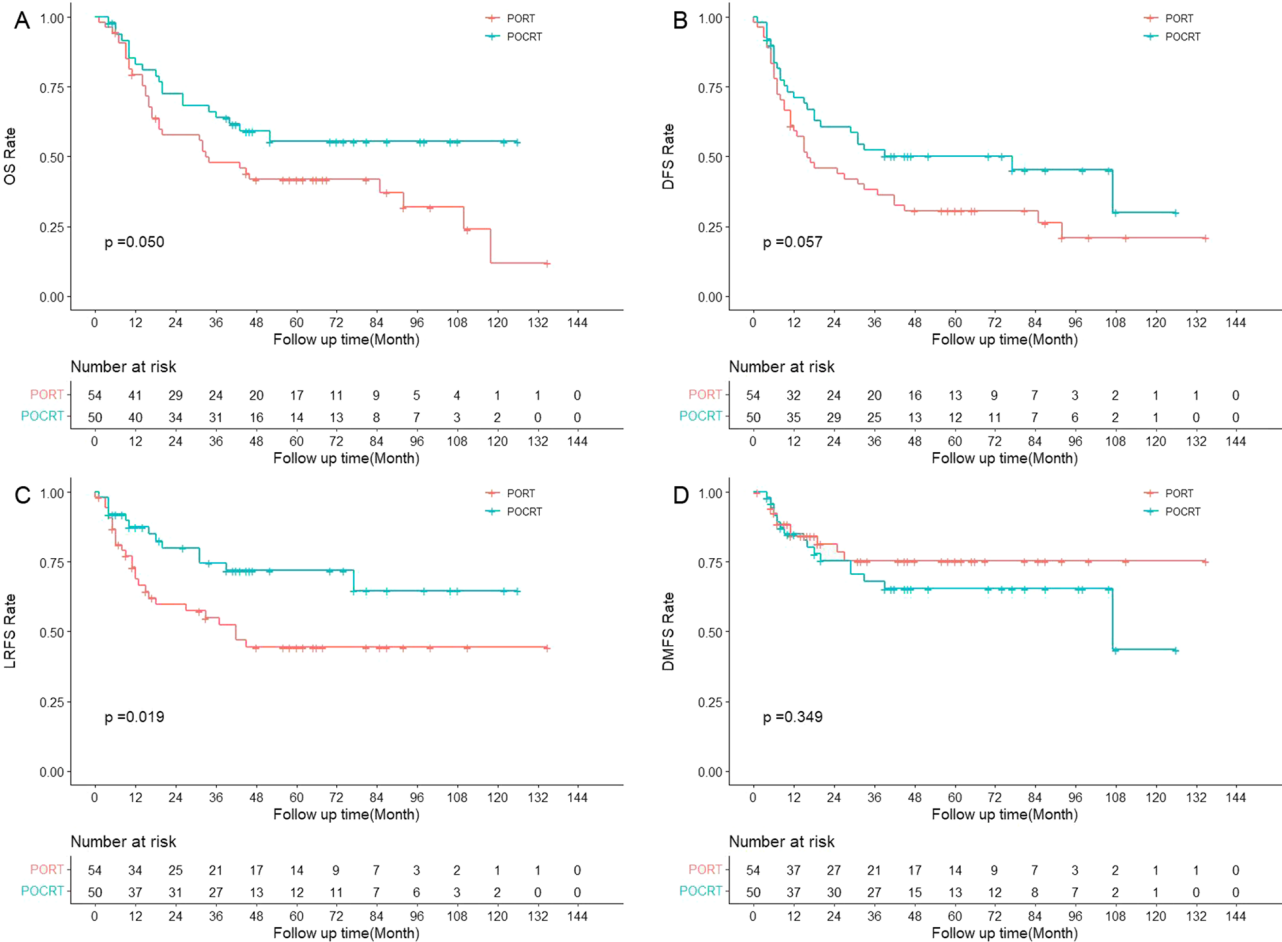
The survival curves of PORT versus POCRT in the matched cohort (N = 104) with pN2-3

**Fig. 5:**
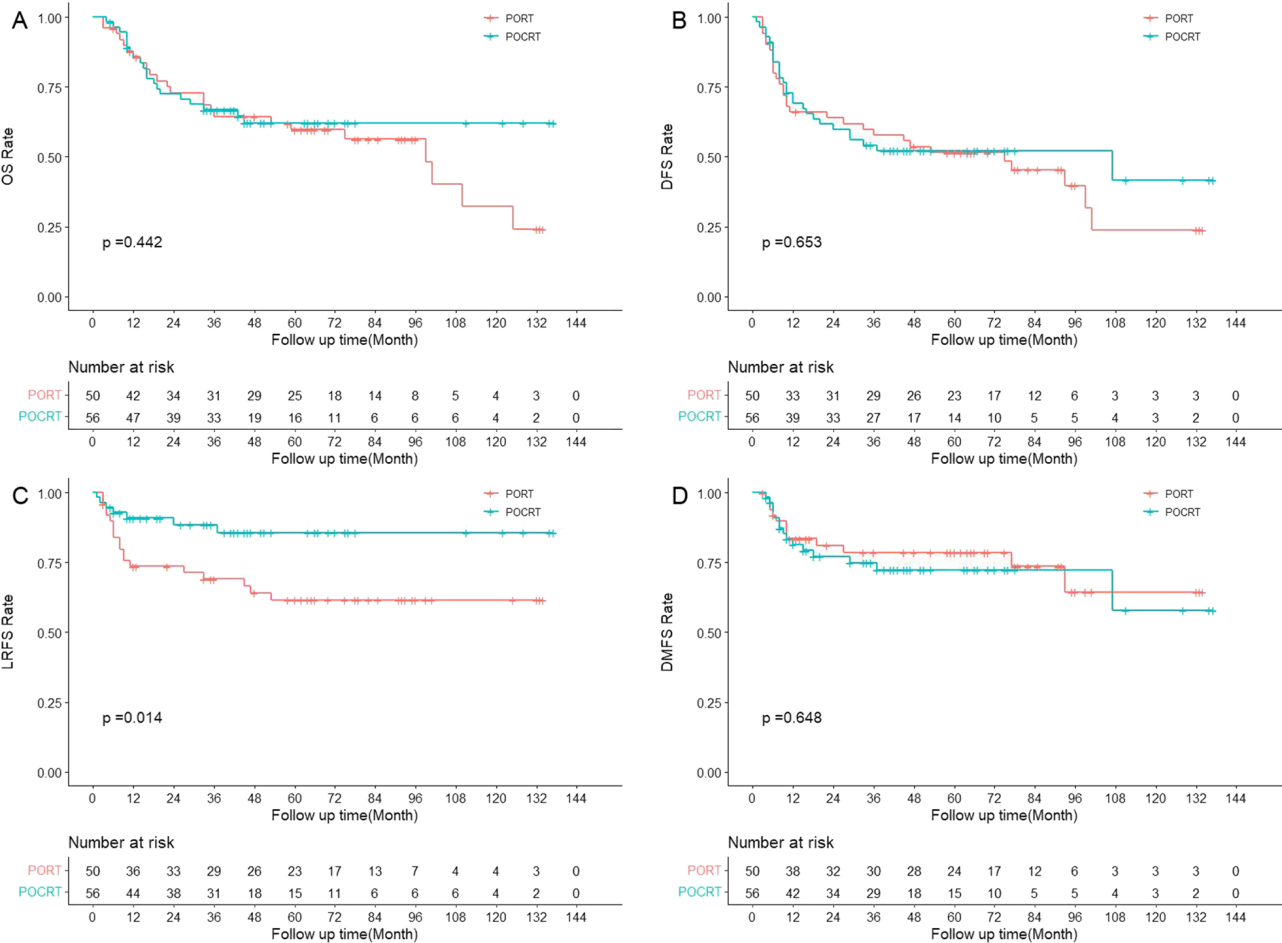
The survival curves of PORT vs. POCRT in the matched cohort (N = 106) with pT3-4

The cohort was divided into different subgroups by four adverse pathological features, including T classification, N classification, PNI and LVI to explore the potential survival benefits of POCRT. First, there was no significant difference in the distribution of baseline characteristics between PORT and POCRT in the subgroup of patients with pT3-4, pN2-3, PNI or LVI, respectively (all *p* > 0.05). As shown in Fig. 4, patients with pN2-3 achieved better survival outcomes from POCRT than PORT in OS (*p* = 0.050, 3-year rate 63.9% vs. 47.9%) and LRFS (*p* = 0.019, 3-year rate 74.6% vs. 54.9%), whereas there was no significant survival improvement in DFS (*p* = 0.057, 3-year rate 52.3% vs. 38.2%) or DMFS (*p* = 0.349, 3-year rate 67.9% vs. 75.3%). Furthermore, POCRT is an independent favorable factor (*p* = 0.023, HR 0.463, 95% CI 0.238–0.901) in patients with pN2-3 for LRFS using multivariate survival analysis. In the subgroup of patients with pT3-4 (shown in Fig. 5), POCRT significantly improved LRFS (*p* = 0.014, 3-year rate 88.5% vs. 69.1%). Meanwhile, in multivariate survival analysis of patients with pT3-4, POCRT was still an independent favorable factor (*p* = 0.009, HR 0.310, 95% CI 0.128–0.747) for LRFS. But there were no significant differences between POCRT and PORT in OS (*p* = 0.442, 3-year rate 66.8% vs. 64.3%), DFS (*p* = 0.653, 3-year rate 54.2% vs. 57.7%) or DMFS (*p* = 0.648, 3-year rate 74.9% vs. 78.5%). POCRT gained similar survival outcomes in OS (*p* = 0.291, 3-year rate 74.1% vs. 65.0%), DFS (*p* = 0.689, 3-year rate 61.0% vs. 60.7%), LRFS (*p* = 0.514, 3-year rate 81.4% vs. 80.4%) and DMFS (*p* = 0.752, 3-year rate 81.3% vs. 83.5%) to PORT in patients with PNI. In the subgroup of patients with LVI, there were no significant differences in OS (*p* = 0.746, 3-year rate 75.5% vs. 71.4%), DFS (*p* = 0.846, 3-year rate 51.9% vs. 57.1%), LRFS (*p* = 0.783, 3-year rate 75.5% vs. 83.9%) or DMFS (*p* = 0.440, 3-year rate 82.1% vs. 69.2%) between POCRT and PORT.

## Discussion

Although pT3-4, pN2-3, PNI and LVI were intermediate-risk features, their effects on the survival of patients with HNSCC were very likely to be covered by the high-risk features of PMs and ECE, considering the strong prognosis. Without the presence of PMs and ECE, we investigated the incidence and the prognostic significance of the four intermediate-risk features. With 41.2% of pT3-4, 38.3% of pN2-3, 21.1% of PNI and 12.8% of LVI, we found that pN2-3 and pT3-4 rather than PNI and LVI were both poor prognostic factors. And fortunately, POCRT could help to improve the survival outcomes of patients with pN2-3 or pT3-4.

The adverse impact of T classification on the prognosis of HNSCC has been reported in previous studies [11, 12]. We also found that different T classifications significantly affected DMFS, but not LRFS or OS. First, patients with an advanced T classification have a higher primary tumor burden, higher risk of micrometastasis and lower DMFS as a result. Meanwhile, surgery and radiotherapy, the main methods of local treatment, were implemented in both treatment groups to reduce the risk of local and regional recurrence. What is more, during the follow-up, patients of two groups with local and regional failure received salvage treatment, which ensured a similar long-term survival. In head and neck cancer, the N classification represents the tumor burden of regional lymph nodes, which is closely related to the prognosis. Our study found that patients with an advanced N classification had poorer local control, shorter survival time and higher risk of distant metastasis. Roberts et al. analyzed 12,437 cases of postoperative head and neck cancer in the Surveillance, Epidemiology, and End Results database from 2004 to 2012 [13], and found that the number of lymph nodes, an indicator of lymph node burden, was also correlated with short OS time. LVI and PNI were reported to be other adverse postoperative pathological features with poor survival outcomes [14–16]; however, the association was not observed in the present study, which was possibly caused by the small sample size of the patients with LVI or PNI.

This study also found that POCRT improved the survival benefit in local control compared to PORT. The possible reason for this is that platinum-based concurrent treatment drugs can increase radiosensitivity to synergistically enhance the cytotoxicity of radiotherapy. This may be achieved by the following mechanisms [17, 18]: (i) inhibiting DNA synthesis; (ii) impeding transcriptional elongation through inter-strand crosslinking; (iii) hindering DNA damage repair; and (iv) promoting the reoxidation of hypoxic cells. We compared two postoperative treatment methods in the pT3-4 subgroup and found that POCRT did improve local control. In patients with pN2-3, POCRT significantly improved LRFS and consequently further improved OS. POCRT increased the survival of patients with pN2-3 or pT3-4, but not all patients with stage III–IV. This was inconsistent with the findings by Trifiletti et al., which showed that POCRT improved OS in all locally advanced HNSCC cases without PM or ECE [8]. Specifically, although T1–2N1M0 is a stage III lesion, patients with this stage had closer survival to those with stage I–II rather than other stage III–IV cancers in this study. Further analysis indicated that 44 patients with T1-2N1M0 obtained similar survival between POCRT and PORT. Therefore, it was clear that patients with pT3-4 or pN2-3 (not all locally advanced lesions), might benefit from POCRT.

There are some limitations in this study. First, the acute and chronic side effects of these two postoperative treatments has not been recorded and compared. The side effects of additional chemotherapy, including hematological, mucous membrane and gastrointestinal adverse events, cannot be ignored [19]. As described in the RTOG 95-01 and EORTC 22931 trials, patients receiving POCRT had more acute grade 3 or 4 adverse events than patients with PORT (*p* < 0.001) [5, 6]. In addition, given the limitations of the retrospective design, we used the PSM method to better reduce the bias and improve statistical performance. All patients in the present study had completed the treatment, therefore efficacy of the study was not affected by incomplete treatment. Further randomized clinical trials are expected to better illustrate the efficacy and safety of POCRT in patients with HNSCC without PMs or ECE. At present, drugs with high efficiency and low toxicity are being explored, such as targeted drugs and immunotherapeutic drugs [20–23].

## Conclusions

In general, this study found that pT3-4 and pN2-3 were independent prognostic factors in intermediate-risk HNSCC without PMs or ECE, whereas PNI and LVI were not. Further analysis found that POCRT improved the survival outcomes of patients with pT3-4 or pN2-3, especially in local tumor control.

## Data Availability

All data generated or analyzed during this study are included in this published article.

## Abbreviations

CI: Confidence interval
CT: Computed tomography
CTV: Clinical target volume
DFS: Disease-free survival
DMFS: Distant metastasis-free survival
ECE: Extracapsular extension
EORTC: European Organization for Research and Treatment of Cancer
HNSCC: Head and neck squamous cell carcinoma
HR: Hazard ratio
LRFS: Locoregional relapse-free survival
LVI: Lymphatic/vascular space invasion
MRI: Magnetic resonance imaging
OS: Overall survival
PM: Positive margin
POCRT: Postoperative chemoradiotherapy
PORT: Postoperative radiotherapy
PNI: Perineural invasion
PSM: Propensity score matching
PTV: Planning target volume
RTOG: Radiation Therapy Oncology Group
